# Early Growth Response Gene-2 (Egr-2) Regulates the Development of B and T Cells

**DOI:** 10.1371/journal.pone.0018498

**Published:** 2011-04-14

**Authors:** Suling Li, Alistair L. J. Symonds, Bo Zhu, Mengya Liu, Meera V. Raymond, Tizong Miao, Ping Wang

**Affiliations:** 1 Department of Biosciences, Brunel University, Uxbridge, London, United Kingdom; 2 Institute of Cell and Molecular Science, Barts and London School of Medicine and Dentistry, University of London, London, United Kingdom; 3 Institute of Cancer, Xinqiao Hospital, Third Military Medical University, Chongqing, People's Republic of China; Institut Pasteur, France

## Abstract

**Background:**

Understanding of how transcription factors are involved in lymphocyte development still remains a challenge. It has been shown that Egr-2 deficiency results in impaired NKT cell development and defective positive selection of T cells. Here we investigated the development of T, B and NKT cells in Egr-2 transgenic mice and the roles in the regulation of distinct stages of B and T cell development.

**Methods and Findings:**

The expression of Egr1, 2 and 3 were analysed at different stages of T and B cell development by RT-PCT and results showed that the expression was strictly regulated at different stages. Forced expression of Egr-2 in CD2^+^ lymphocytes resulted in a severe reduction of CD4^+^CD8^+^ (DP) cells in thymus and pro-B cells in bone marrow, which was associated with reduced expression of Notch1 in ISP thymocytes and Pax5 in pro-B cells, suggesting that retraction of Egr-2 at the ISP and pro-B cell stages is important for the activation of lineage differentiation programs. In contrast to reduction of DP and pro-B cells, Egr-2 enhanced the maturation of DP cells into single positive (SP) T and NKT cells in thymus, and immature B cells into mature B cells in bone marrow.

**Conclusions:**

Our results demonstrate that Egr-2 expressed in restricted stages of lymphocyte development plays a dynamic, but similar role for the development of T, NKT and B cells.

## Introduction

T and B cell development in the thymus or bone marrow is a strictly controlled process that requires the precise alternation of gene expression programs orchestrated by specific transcription factors [1], [2]. T and B cells initially develop from lymphoid-primed multipotent progenitors (LMPP) [3]. The current paradigm is that as the cells develop, the changes induced by newly synthesized transcription factors dictate lineage differentiation and maturation of T or B cells [3]. Although some major transcription factors have been defined for lineage commitment, such as EBF1 and Pax5 for B cells and Notch1 for T cells, the transcription factors that are specifically involved in the regulation of distinct stages of B and T cell development are largely unknown [1], [2].

Egr-1 and Egr-3 induced by pre-TCR signaling have been found to be important for the DN to DP transition [4], [5], [6], [7], [8], [9], [10]. Among the four members of the Egr family, Egr-1, -2 and -3 are expressed during thymocyte development [4], [5]. Overexpression of Egr-1 or Egr-3 in pre-TCR deficient mice partially restores the progression through the b-selection check point [4], [7], [11], [12], and induces downregulation of Rag1, Rag2 and pTCRa, resembling some characteristics of the endogenous effects of pre-TCR signaling. Mice deficient in Egr-1 or in Egr-2 have normal β-selection, but a defect in positive selection [6], [10], [13], while Egr-3 deficient thymocytes have a partial block at the DN3 stage that is largely due to defective proliferation of DN4 cells [7]. Interestingly, mice lacking both Egr-1 and Egr-3 had a severe reduction of thymocyte numbers, with reduction of the DN4/DN3 ratio [14]. The low thymic cellularity is due to massive apoptosis of thymocytes resulting from an autonomous survival defect [14], which has not been seen in single knockout models [7], [6], suggesting a redundant function between these two Egr molecules. A similar function of Egr-1 and Egr-3 in thymocyte development is further supported by an identical phenotype in Egr-1 and Egr-3 transgenic models [8], [11].

Like Egr-1 and -3, Egr-2 has also been found to be induced by pre-TCR signaling in DN3 thymocytes [4]. Overexpression of Egr-2 in vitro induces downregulation of Rag1 and Rag2 gene expression [4], indicating an overlapping function between Egr-2 and Egr-1 and Egr-3. In an adoptive transfer model with fetal liver cells from Egr-2 KO mice, the reconstituted thymocytes in Rag2−/− mice have normal development of conventional T cells, but a defect in NKT cell development [15]. We have recently developed a CD2-specific Egr-2 KO model and did not find changes in T cell development [16]. To investigate further the function of Egr-2 in the development of lymphocytes, we established CD2-specific Egr-2 transgenic (Egr-2 cTg) mice. We discovered a defect in thymocyte development in Egr-2 cTg mice which was remarkably similar to that in Egr-1 and Egr-3 transgenic mice [8], [11]. However, we discovered two important novel functions of Egr-2; enhancement of DP cell maturation into SP and NKT cells, and regulation of B cell development. Intriguingly, the defects in B cell development observed in Egr-2 cTg mice resembled those seen in thymocytes in these mice suggesting that Egr-2 may have a similar mechanism of action in these two cell types. The defects in thymocyte and B cell development are accompanied by reduced expression of lineage regulators, Notch1 in ISP thymocytes and Pax5 in Pro-B cells in Egr-2 cTg. Our results demonstrate a general function of Egr-2 in regulation of lymphocyte development, suggesting that Egr-2 expressed in restricted stages of lymphocyte development plays a dynamic role for the regulation of progenitors and the maturation of lymphocytes.

## Materials and Methods

### Mice

Egr-2 cKO mice, a line with deletion of Egr-2 in CD2^+^ lymphocytes on a B6 background, were described in our previous publication [16]. B6 mice were purchased from Harlan UK Ltd. (Oxon, England). To generate Egr-2 transgenic mice, the Egr-2 coding region, and flanking EcoRI site, was amplified by PCR with primers (sense TGACGAATTCATGATGACCGCCAAGGC and anti-sense CACACCCTAACTGACACACATTCC) from an Egr-2 expression construct containing Egr-2 cDNA [16]. The PCR product was cloned into the hu-CD2 vector containing the human CD-2 promoter [17]. A vector free DNA fragment containing the human CD-2 promoter, hCD2 LCR, Egr-2 cDNA and poly-A signal was injected into pronuclei of fertilized oocytes from C57BL/6xCD1 F1 mice by the transgenic core facility at Barts and The London Medical School, Queen Mary University of London. Transgenic founders were detected by PCR analysis of genomic tail DNA with primers: sense CCACCAGTCTCACTTCAGTTCC and anti-sense CAGCTGGTGCATAAAACCACTG. The transgenic lines were propagated by sequential crossing to C57BL/6 mice. Two transgenic founder lines, Tg14 and Tg18, both expressing high levels of the Egr-2 transgene and displaying similar phenotypes, were derived from C57BL/6. All mice were maintained in the Biological Services Unit, Barts and The London School of Medicine, and used according to established institutional guidelines under the authority of a UK Home Office project licence (Guidance on the Operation of Animals, Scientific Procedures Act 1986). The Tg14 line was used for most of the experiments and termed Egr-2 cTg.

### Flow cytometry and antibodies

Flourescein isothiocyanate (FITC)-conjugated antibodies to B220, CD4, CD8 and TCRβ; phycoerythrin (PE)-conjugated antibodies to CD3, CD4, CD5, CD8, CD19, CD25, CD43, CD127, IgM and NK1.1; PerCP labelled antibody to CD3, NK1.1, CD19, B220; allophycocyanin (APC)-conjugated antibodies to TCRβ, NK1.1, CD43, CD44, CD3, CD19 and APC-conjugated Annexin V were obtained from BD Biosciences. PE-conjugated pre-loaded CD1 tetramer was from ProImmune.

Single cell suspensions were prepared from 6–10 wk old mice. For staining, 2×10^6^ cells were stained with optimal amounts of fluorochrome-labelled antibodies and analyzed on a LSRII (BD Immunocytometry Systems) and the data were analyzed using FlowJo (Tree Star). Cell sorting was performed on a FACSAria sorter with DIVA option (BD Immunocytometry Systems).

### Quantitative real-time PCR

Real time PCR was carried out as described [16]. Briefly, total RNA was extracted from total thymocytes or FACS sorted DN1, DN2, DN3, DN4, DP or ISP cells and total bone marrow cells or FACS sorted pre-pro-B, pro-B, pre-B, immature-B and mature-B cells, using TRIzol (Invitrogen Life Technologies), and was reverse transcribed using oligo(dT) primers (Amersham Biosciences). Quantitative real-time PCR was performed on a Rotor-Gene system (Corbett Robotics) using SYBR green PCR master mix (Qiagen). The primers used in quantification of gene expression were; Egr-2; sense 5′-CTTCAGCCGAAGTGACCACC-3′; antisense 5′-GCTCTTCCGTTCCTTCTGCC-3′, E2F2; sense 5-GTAGAAGAGGGTGTGACAGC-3; antisense 5-AGCACTGACTCTGGGATCGC-3, Bcl2; sense 5-GGAGAAATCAAACAGAGGTCGC-3; antisense 5-CGTCAACAGGGAGATGTCACC-3, BclXL; sense 5-CATTGTTCCCGTAGAGATCC-3; antisense 5-TTCGGGATGGAGTAAACTGG-3, c-MYB; sense 5-GTTCTTAGGTACGGTAAAGGC-3 antisense 5-TTCTTCTGCTCAAACCACTGG-3, c-MYC; sense 5-AGGGGTTTGCCTCTTCTCCAC-3 antisense 5-TTCTCTCCTTCCTCGGACTCG-3, RORgt sense 5-CCGCTGAGAGGGCTTCAC-3; antisense 5-TGCAGGAGTAGGCCACATTACA-3, Notch1 sense 5- CAGCTTGCACAACCAGACAGAC-3, anti-sense 5-ACGGAGTACGGCCCATGTT-3, TCF1 sense 5-TGCTGTCTATATCCGCAGGAAG-3, anti-sense 5-CGATCTCTCTGGATTTTATTCTCT-3, Gata2 sense 5-TGCAACACACCACCCGATACC-3, antisense 5-CAATTTGCACAACAGGTGCCC-3, Gata3 sense 5-GAGGTGGTTGTCTGC-3, antisense 5- TTTCACAGCACTAGAGACCCTGTTA-3, Runx1 sense 5-CCTGCTTGGGTGTGAGGCCC-3, antisense 5-GCCTCGCTCATCTTGCCGGG-3, β-actin sense 5-AATCGTGCGTGACATCAAAG-3, antisense 5-ATGCCACAGGATTCCATACC-3, ICER sense 5-GCTAGTTGGTACTGCCATGGTAGC-3, antisense 5-AGCCCAACATGGCTGTAACTGGAG-3, Nor1 sense 5-AGGATACACTTCCTGTGTCAAGGG-3, antisense 5-CCATTTCATAGCATGACTGCCTCC-3, Nurr1 sense 5-GCAGTTGCTTGACACGCAGGTGCC-3, antisense 5-CCTGTGGGCTCTTCGGCTTCGAGG-3, Nur77 sense 5-GCTATTCCATGCCAGCAGCTTTCC-3, antisense 5-CCGTACAACTTCCTTCACCATGCC-3, Rag1 sense 5-ACCTGAGATCGATGATCCCACATCT-3, antisense 5-CTGTCGATTCTCGAGTATGGAAGTC-3, Rag2 sense 5-AGACCCGTTTGAGCTACTTCTTGC-3, antisense 5-GGAGCTTCCTTCTCTATGAAGTCG-3, Egr1 sense 5-ACGACAGCAGTCCCATCTACTCGG-3, antisense 5-GGACTCGACAGGGCAAGCATATGG-3, LTα sense 5-GTCTGGTTCTCCACATGACACTGC-3, antisense 5-GAAAAGAGCTGGACCTCGTGTGCC-3, Egr3 sense 5-CGACTCGGTAGCCCATTACAATCAGA-3, antisense 5-GAGATCGCCGCAGTTGGAATAAGGAG-3, LTβ sense 5-TGCCTATCACTGTCCTGGCTGTGC-3, antisense 5-AACGCTTCTTCTTGGCTCGCCTCC-3, LTβR sense 5-CTACCCATGTCTGGAGACTTGTCC-3, antisense 5-CTGTCAGAGGTCTTGGCATCCTAG-3, LIGHT sense 5-TCAGCTGCTCTGGCATGGAGAGTG-3, antisense 5-TGCTGGGTTGGCCTGGTGAGATCG-3, AIRE sense 5-GATGTGGACCTAAACCAGTCCCGG-3, antisense 5-CTGGCTTTAGGCTGCTACCACTCC-3, Pax5 sense 5-CCA TCA GGA CAG GAC ATG GAG-3, antisense 5-GGC AAG TTC CAC TAT CCT TTG-3. LEF-1 sense 5- TCCCTAGCCACAGTCCAATA-3, anti-sense 5- AGCAACAGGCTTAGAACGTG-3, Id1 sense 5-TACTTGGTCTGTCGGAGCAA-3, antisense 5-GATCAAACCCTCTACCCACT-3, Id2 sense 5-CCTGGACTCGCATCCCACTA-3, antisense 5-TGCTATCATTCGACATAAGCTCAGA-3, Id3 sense 5-ATCCTGCAGCGTGTCATAGACT-3, antisense 5-AGGCGTTGAGTTCAGGGTAAGT-3, HEB sense 5-AAATCAGATGATGAGTCCTCCC-3, anti-sense 5-CTCTGGAACTGGCTGATGTTT-3, E2A sense 5-GCATAGGAAGCTCAGCAGAGA-3, anti-sense 5-AAGCATACAGGACTGCAAGGAG-3.

The data were analyzed using the Rotor-Gene Software. All samples were run in duplicate, and relative mRNA expression levels were obtained by normalizing against the level of β-actin from the same sample under the same program using: relative expression = 2(CTβ-actin-CTtarget)×10,000.

### TUNEL assay

Anesthetized mice received intracardiac PBS followed by aldehyde perfusion (4% paraformaldehyde in PBS). The thymus were removed and frozen in OCT embedding medium (VWR) and stored in liquid nitrogen. TUNEL assay was performed with TACS™ TdT Kit (Roche Diagnostics GmbH, Roche Applied Science, Mannheim, Germany) as per the manufacturer's protocol. Briefly, cryosections were fixed with 3.7% formaldehyde solution, to prevent the loss of low molecular weight DNA fragments, and then the cell membranes were permeabilized with Proteinase K. Apoptotic cells were then labelled in situ by addition of biotinylated nucleotides onto the 3-OH ends of the DNA fragments by Terminal deoxynucleotidyl Transferase (TdT). The biotinylated nucleotides were detected using a streptavidin-fluorescein conjugate. The fluorescein-stained cells were subsequently visualized using a fluorescence microscope.

### Statistics

The Mann-Whitney U test was used. The normality of data distribution and the homogeneity of variances of the data were verified by the K-S Lilliefors and Cochran C tests, respectively.

## Results

### Sustained expression of Egr-2 affects thymocyte development

**Figure 1:**
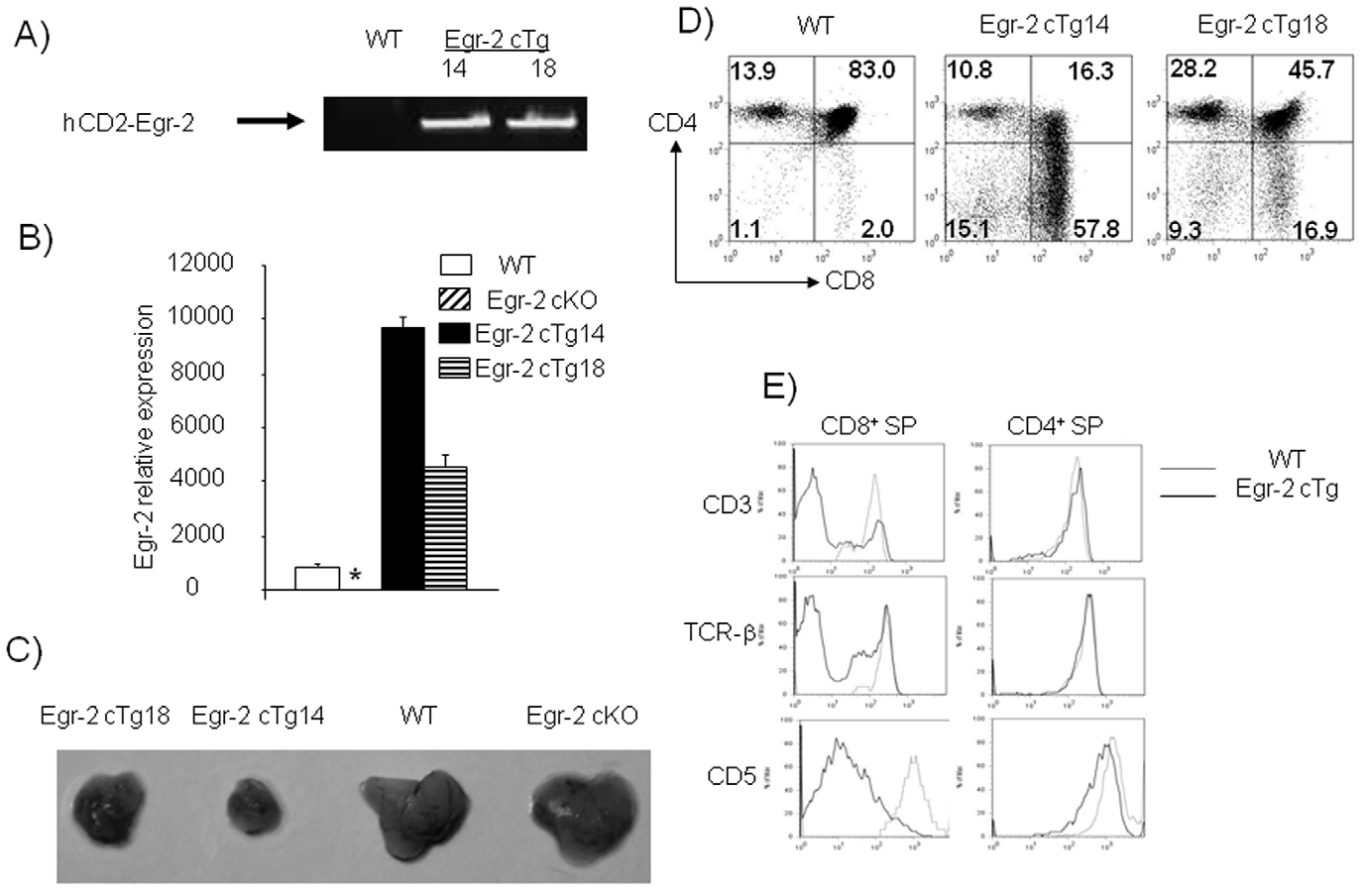
Defective thymus development in Egr-2 transgenic mice. A). confirmation of presence of transgene by PCR. B). expression of Egr-2 in thymocytes from wild type, Egr-2 cKO and two Egr-2 cTg mouse lines (14 and 18) determined by quantitative RT-PCR. The relative expression was measured after normalization against b-actin. _*_ Egr-2 was not detected in Egr-2 cKO T cells. C). Thymus size in wild type, Egr-2 cTg and Egr-2 cKO mice. D). Thymocytes from wild type and Egr-2 transgenic mice were stained with anti-CD4 and anti-CD8. E). Expression of CD3, TCRβ and CD5 by gated CD4^+^ or CD8^+^SP T cells in thymus. The data are representative of three experiments in which 3 to 5 mice from each group were used.

Despite expression of Egr-2 in thymocytes, deletion of Egr-2 in CD2^+^ lymphocytes (Egr-2 cKO) did not alter the development of thymocytes [15] and [16]. To further analyze the function of Egr-2 in lymphocytes, two CD2-specific Egr-2 transgenic (Egr-2 cTg) lines were established (Fig. 1A and B).

**Figure 2:**
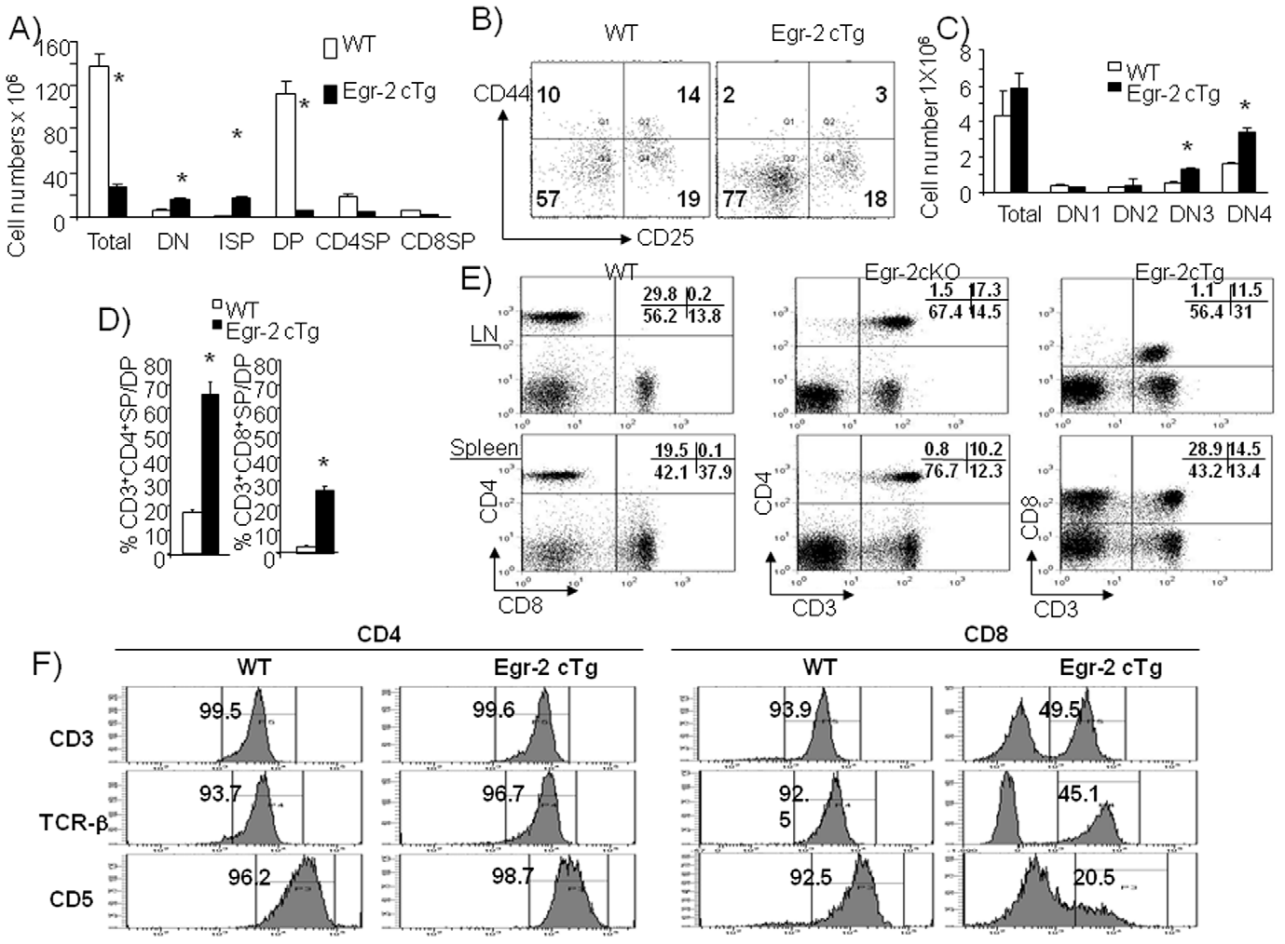
Development of thymocytes. A). Absolute number of thymocyte subpopulations in thymus. The ISP cells were defined as CD4^−^CD8^+^CD3^−^ (see Fig. 1E). B). The subpopulations of DN cells in thymus were examined by analysis of CD25 and CD44 expression on gated CD4^−^CD8^−^ DN cells. C). Absolute number of DN subsets in thymus. D). Percentage of CD3^+^DP cells that develop into CD3^+^CD4^+^ or CD3^+^CD8^+^ cells in thymus. E). Subpopulations of T cells in lymph nodes and spleen. F). Expression of CD3, TCRβ and CD5 on CD4^+^ or CD8^+^ cells in spleens. * p<0.001. The data are representative of more than three experiments in which 3 to 5 mice from each group were used.

**Figure 3:**
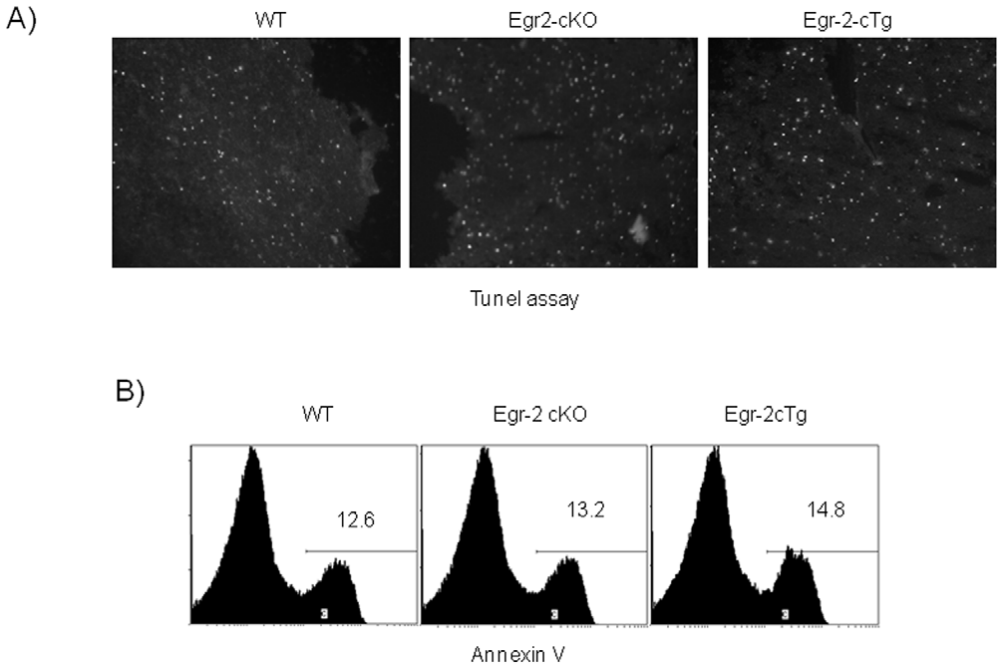
Thymocyte apoptosis. A). Tunel assay on thymus of wild type, Egr-2 cTg and Egr-2 cKO mice. B). Percentage of Annexin V^+^ and PI^−^ thymocytes in wild type, Egr-2 cTg and Egr-2cKO mice. The data are representative of three to five mice.

The size of the thymus in Egr-2 cTg mice was only one third of wild type mice (Fig. 1C) with increased CD4^−^CD8^−^ DN and CD4^−^CD8^+^CD3^−^ ISP, but severely reduced CD4^+^CD8^+^ DP cells (Fig. 1 D, E, Fig. 2 A). Although the changes in percentages of thymocyte subsets were strikingly similar to those in Egr-1 and Egr-3 transgenic mice [8], [11], the changes in absolute number of DN subsets differed [8], [12]. In contrast to the normal number of DN1 and DN2 cells, the absolute number of DN3 and DN4 cells in Egr-2 cTg mice were double that of wild type mice (Fig. 2 B and C) despite a similar level of Egr-2 transgene expression (Figure S1). Interestingly, in contrast to the reduction of DP cells in the thymus, the percentage of DP cells that matured into SP cells was increased (Fig. 2D). The T cell subsets in the periphery were also altered and resembled that seen in the thymus (Fig. 2E, F). Although Egr-2 has been found to induce expression of FasL on activated T cells [18], the expression of FasL was normal (data not shown) and a normal apoptosis was detected in Egr-2 cKO and Egr-2 cTg thymus (Fig. 3A and B). We investigated the proximal signalling of T cell receptor and proliferation of thymocytes following stimulation with anti-CD3 in vitro and a similar result was found in thymocytes from wild type and Egr-2 cTg mice (Figure S2). These results suggest that Egr-2 plays different roles at the early and late stages of thymocyte development and the function of Egr-2 may not associate with cell death and TCR signalling.

### Forced expression of Egr-2 enhances NKT cell development

**Figure 4:**
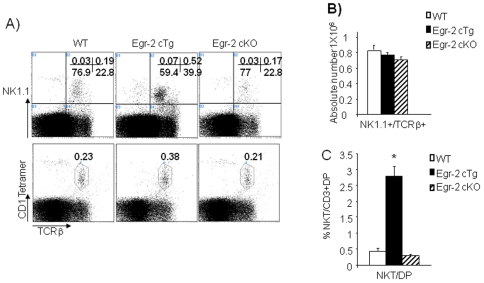
Development of NKT cells in thymus. A). Thymocytes from wild type, Egr-2 cKO and Egr-2 cTg mice were stained with NK1.1 and TCRβ antibodies (top) or with TCRvβ and CD1 tetramer (bottom). B). Absolute numbers of NKT cells in thymus of Egr-2 cKO, Egr-2 cTg and wild type mice. C). Percentages of DP cells that develop into NKT cells in thymus of Egr-2 cKO, Egr-2 cTg and wild type mice. * p<0.001. The data are representative of more than two experiments in which 3 to 5 mice from each group were used.

NKT cells develop from DP cells and it has been found recently that Egr-2 induced by NFAT is important for NKT cell development [15]. We found that the development of NKT cells, detected as NK1.1^+^TCRβ^+^ or CD1 tetramer^+^TCRβ^+^, in Egr-2 cKO mice was comparable to that in wild type mice, while increased in Egr-2 cTg mice (Fig. 4A). Despite the severe reduction in the DP population, the absolute number of NKT cells in the thymus of Egr-2 cTg mice was similar to that in wild type mice (Fig. 4B). Therefore, the ratio of NKT/DP cells in Egr-2 cTg mice was significantly increased (Fig. 4C) suggesting that in addition to the enhanced maturation of DP cells into SP T cells, Egr-2 enhances the maturation of DP cells into NKT cells. Our results suggest that Egr-2 supports the maturation of DP cells to T or NKT cells.

### Sustained Egr-2 expression blocks the development of pre-pro-B cells into pro-B cells, but enhances B cell maturation

**Figure 5:**
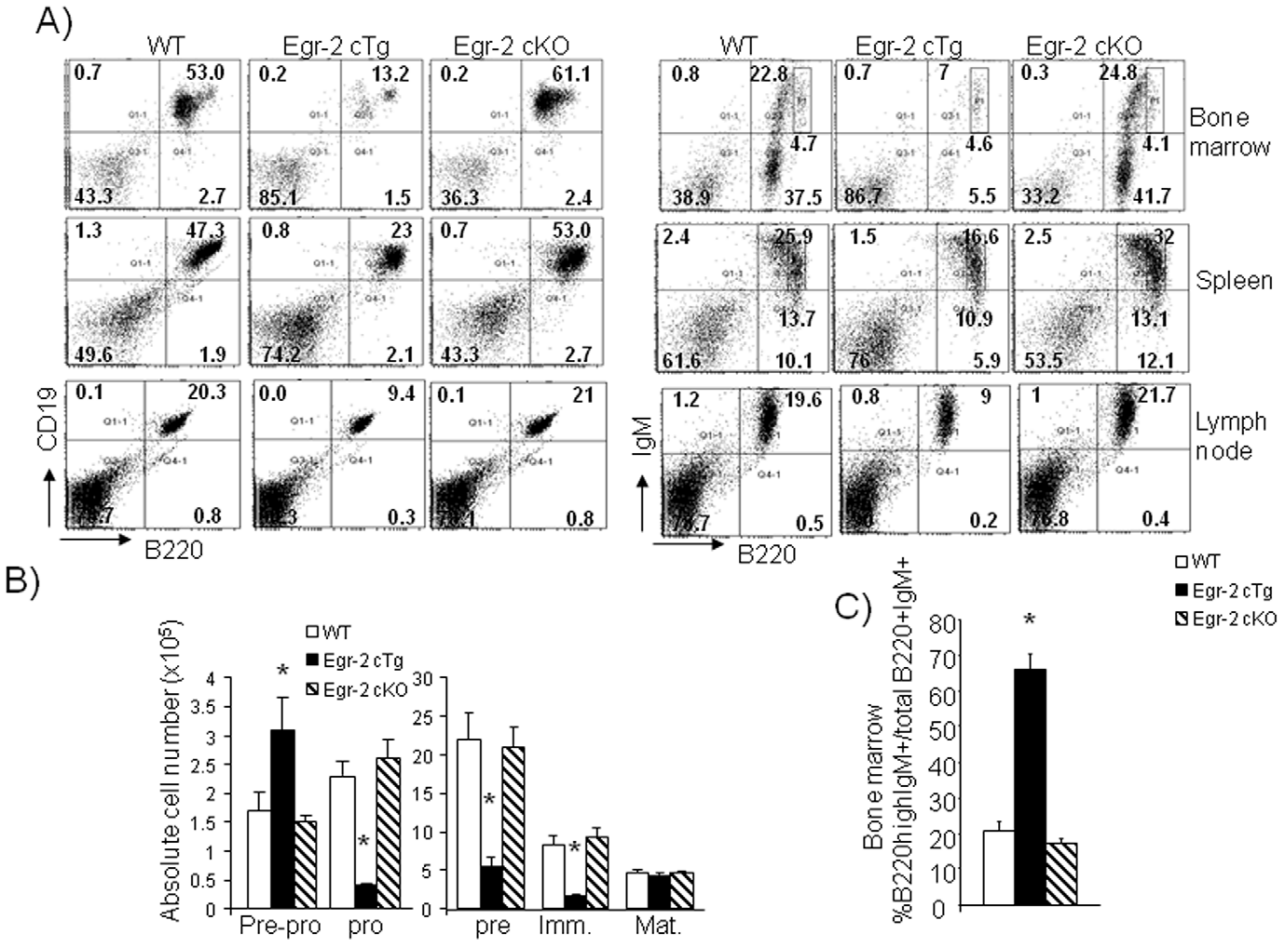
B cell development. A). Cells from bone marrow, lymph nodes and spleen of wild type, Egr-2 cKO and Egr-2 cTg mice were co-stained with anti-CD19 and anti-B220 (left) or anti-IgM and anti-B220 (right). B). Absolute number of B cell precursor subpopulations in bone marrow of wild type, Egr-2 cKO and Egr-2 cTg mice. Pre-pro-B cells defined as NK1.1^−^B220^+^CD19^−^CD43^+^, pro-B cells defined as NK1.1^−^B220^+^CD19^+^CD43^+^, pre-B cells as NK1.1^−^B220^+^CD43^−^, immature B cells as NK1.1^−^B220^+^IgM^+^ and mature B cells defined as NK1.1-B220highIgM+. C). Percentages of immature B cells that develop into mature B cells in bone marrow. * p<0.001. The data are representative of more than two experiments in which 3 to 5 mice from each group were used.

It is unknown whether Egr-2 is involved in B cell development. The CD2 promoter used to create Egr-2 cKO and cTg mice is active in both T and B lymphocytes [17]. Therefore, B cell development was also examined in these mice. Similar to thymocyte development, the development of B cells in the bone marrow was normal in Egr-2 cKO mice, but severely defective in Egr-2 cTg mice (Fig. 5). Subsequently, a reduction of B cells in the peripheral lymphoid organs of Egr-2 cTg mice was detected (Fig. 5A). In the bone marrow pre-pro-B cells, defined by a NK1.1^−^B220^+^CD19^−^CD43^+^ phenotype, were slightly increased in Egr-2 cTg mice, while pro-B cells, defined by NK1.1^−^B220^+^CD19^+^CD43^+^, pre-B cells, NK1.1^−^B220^+^CD43^−^, and immature-B cells, NK1.1^−^B220^+^IgM^+^, were severely reduced (Fig. 5B). However, in contrast to the severe defects in pro-, pre and immature B cells, the number of mature B cells, defined by NK1.1^−^B220highIgM^+^, in the bone marrow in Egr-2 cTg mice was normal (Fig. 5A and B). Therefore, similar to the enhanced maturation of DP cells into SP and NKT cells in the thymus, the ratio of mature/immature B cells in the bone marrow showed that more than 60% of immature B cells developed into mature B cells while only 20% did in wild type mice (Fig. 5C). The levels of Egr-2 transgene expression were similar in different stages of B cells (Figure S1). Thus, we have discovered that Egr-2 does not specifically function in the development of the T cell lineage, but also in B cells and has a similar effect in both lineages.

### Expression of Egr molecules is modulated during lymphocyte development

**Figure 6:**
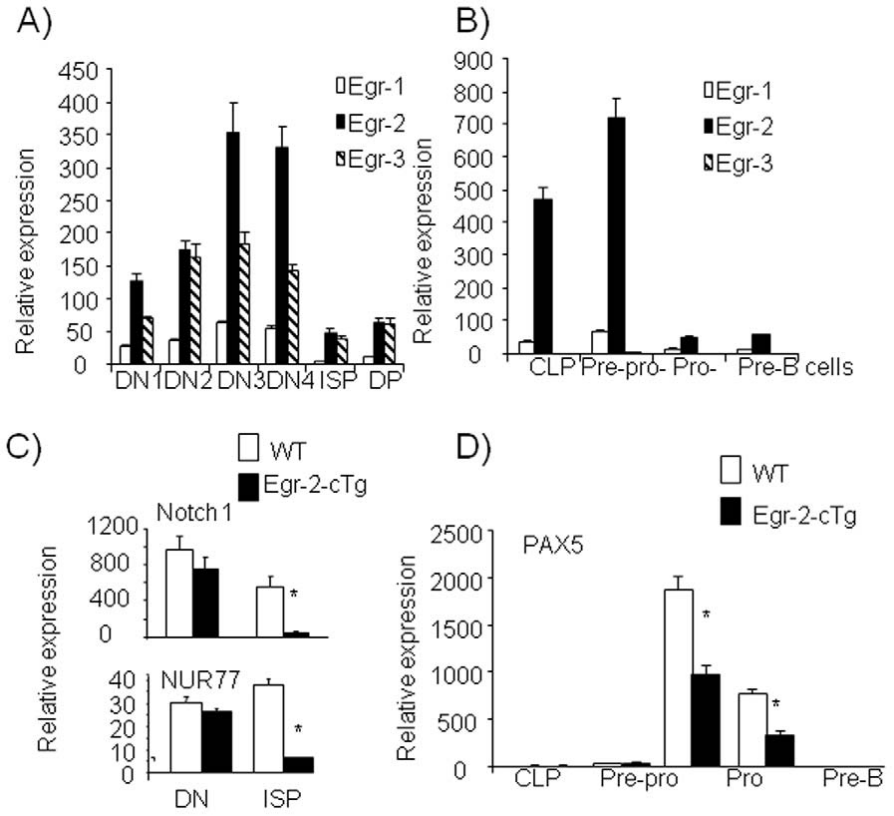
Kinetic expression of Egr in wild type and expression of regulatory genes in thymocytes and bone marrow B cell precursors from Egr-2 cTg mice. Egr expression as determined by quantitative RT-PCR in subpopulations of thymocytes (A) or bone marrow B cell precursors (B) of wild type mice. CD8^+^CD4^−^CD3^−^ ISP cells and CD4^+^CD8^+^ DP cells were sorted by FACS. DN cell subpopulations were isolated based on their expression of CD25 and CD44 after first gating on CD4^−^CD8^−^ cells. B cell subsets were sorted by FACS based on triple staining with CD19^−^B220^−^CD127^+^ for CLP cells, NK1.1^−^B220^+^CD19^−^CD43^+^ for pre-pro-B cells, NK1.1^−^B220^+^CD19^+^CD43^+^ for pro-B cells and NK1.1^−^B220^+^CD43^−^ for pre-B cells. C). Expression of Notch1 and NUR77 genes in DN (CD4^−^CD8^−^) and ISP (CD8^+^CD3^−^) thymocytes from wild type and Egr-2 cTg mice determined by quantitative RT-PCR. D). Expression of Pax5 in sub-populations of B cells from bone marrow of wild type and Egr-2 cTg mice. The relative expression is shown as mean ± s.e.m. for duplicate determinations, relative to actin. * p<0.001. The data are representative of more than two experiments in which 3 to 5 mice from each group were used.

Previous reports show that Egr-1, -2 and -3 are expressed in DN cells [4]. The defects in thymocyte and B cell differentiation in Egr-2 cTg mice suggest that Egr expression is strictly modulated at different stages of thymocyte and B cell development. Therefore, the expression of Egr-1, -2 and -3 was analyzed in thymocytes and bone marrow B cells from wild type mice at different stages. Egr-2 and Egr-3 were expressed at relatively high levels while the levels of Egr-1 were much lower at all stages of DN cells in the thymus (Fig. 6A), while Egr-2 was significantly higher than Egr-1 and Egr-3 in B220^−^CD19^−^CD127^+^ common lymphoid progenitors (CLP) and pre-pro-B cells (Fig. 6B). However, the expression profile of these three Egr molecules across the different stages was essentially identical. They were all expressed in DN2, DN3 and DN4 thymocytes as well as CLP and pre-pro-B cells, while the expression was minimal in ISP and pro-B cells (Fig. 6A and B). The comparable expression profiles correlate well with the similar defects in DP thymocytes in all three Egr transgenic mice [8], [11] and taken together provide strong evidence for a redundant role of these molecules. Importantly, these results indicate that the silencing of Egr expression at the ISP and pro-B cell stages is critical for the differentiation of thymocytes and B cells.

### Over-expression of Egr-2 impairs Notch1 and Nur77 expression in ISP thymocytes and Pax5 in pro-B cells

Lymphocyte development consists of an irreversible progression of cells through distinct developmental stages which is regulated by the coordinated expression of a multitude of transcription factors [1], [2], [19]. We have now shown that Egr molecules are expressed in the early stages of T and B cell development and that the expression was reduced at the ISP thymocyte and pro-B cell stages. Egr-1 has been found to induce expression of Id3, a helix-loop-helix protein [20]. Together with E2A, Id proteins are important for the early development of B and T lymphocytes [21]. To investigate the possible role of Egr-2 in the expression of regulatory genes in thymocyte and/or B cell development, we analyzed the expression of several well characterized regulatory genes, including E2F2, Bcl2, BclXL, E2A, Id1, Id2, Id3, MYB, c-Myc, RORgt, Gata2, Gata3, Runx1, ICER, AIRE, TCF1, LEF-1, HEB, Notch1, Nurr1, NUR77, PAX5 and LEF-1 [1], [19], in DN and ISP thymocytes and pro-B cells from Egr-2 cTg and wild type mice. The expression of most of the genes tested was unchanged (data not shown). However, the expression of Notch1 was reduced in ISP cells from Egr-2 cTg mice (Fig. 6C). In addition to Notch1, the expression of the orphan nuclear receptor Nur77 was defective in ISP cells from Egr-2 cTg mice (Fig. 6C). Interestingly, the expression of Pax5, which is required for B cell lineage commitment and differentiation, was reduced in pro-B cells from Egr-2 cTg mice (Fig. 6D). These results suggest that withdrawal of Egr expression at the early stages of lymphocyte development is important for the final development of T and B cells.

## Discussion

Egr-2 has been found to be important for NKT cell development and positive selection of thymocytes in separate studies [10], [13], [15]. We have now shown that together with Egr-1 and Egr-3, Egr-2 is expressed only in selected stages of T and B cell development and is repressed in ISP thymocytes and pro-B cells. Forced expression of Egr-2 severely impairs the development of DP cells in thymus and immature B cells in bone marrow, respectively. In contrast to the defects in precursor development in Egr-2 cTg mice, the percentage of DP cells developed into SP and NKT cells and the percentage of immature B cells into mature B cells were increased, suggesting that Egr-2 has a general role in the regulation of progenitors and terminal maturation of lymphocytes, but at restricted stages.

Expression of Egr-1, -2 and -3 molecules can be induced in DN cells upon pre-TCR stimulation [4]. The involvement of Egr proteins in β-selection is demonstrated by the fact that overexpression of Egr-1 or Egr-3 can overcome the deficiency in β-selection resulting from Rag deficiency and drives the development of cells to the ISP stage [7], [11]. However, the most significant and common phenotype displayed in transgenic models of Egr-1, -2 and -3 is the reduction of the DP population and the increase of ISP cells [8], [11], which suggests that Egr molecules are important for the development of DN cells following β-selection and withdraw of Egr-2 expression after β-selection is important for the differentiation of ISP cells to DP cells. Although Egr-1 or Egr-2 deficiency does not affect thymocyte development significantly [6], [10], [16], Egr-3 deficiency results in thymic atrophy due to impaired proliferation after β-selection, but the DN to DP transition is normal [7], indicating an important function of Egr molecules in supporting expansion and survival of thymocytes after β-selection. The defects in proliferation and survival of thymocytes were much more severe in mice lacking both Egr-1 and Egr-3 and observed in both DN and DP subsets suggesting a survival function of Egr molecules at early and late stages of thymocyte development [14], which is directly supported by our findings that DN cells and percentage of DP differentiated to SP and NKT cells are increased in Egr-2 cTg mice. A response of transient Egr-3 expression to pre-TCR stimulation has been suggested to be important in order to allow later induction of Bcl-XL and RORgt since sustained expression of Egr-3 decreases the expression of Bcl-XL and RORgt [8], [9]: important factors for the survival of mature thymocytes during selection [1]. Therefore, overlapping with Egr-1 and -2, the regulated expression of Egr-3 in response to pre-TCR stimulation is important for the maturation of DP cells. The increased maturation of DP cells into SP and NKT cells in Egr-2 cTg mice indirectly supports the findings that Egr-2 is important for positive selection and the development of NKT cells [10], [13], [15].

Our results extend the function of Egr-2 to the development of B cells and indicate a similar mechanism for the modulation of Egr-2 expression and the function of Egr-2 in regulation of B cell progenitors and B cell maturation. The expression of Egr-1 can be induced in pre-, immature- and mature-B cells by stimulation with anti-IgM [22]. However, the expression of Egr in B cell progenitors is unknown. We have now demonstrated that Egr-1, -2 and -3 are expressed in CLP and pre-pro-B cells and, importantly, the expression is repressed in pro-B cells, a similar expression pattern to that seen in DN and ISP thymocytes. In addition to the similar expression pattern, the pattern of defects is also similar between B and T cell development in Egr-2 cTg mice, as demonstrated by a severe reduction of pro-B cells and enhanced terminal B cell maturation, suggesting a common mechanism operated by Egr-2 in both lineages. Despite the severe defect of pro-B cells in Egr-2 cTg mice, the development of B cells in Egr-2 cKO mice is normal. This may be due to the weak activity of the CD2 promoter in B cells as shown previously or perhaps due to the redundant function of Egr-1 and Egr-3 [14].

The normal expression of the major molecules involved in cell death and survival (data not shown) and the normal apoptosis of thymocytes in the thymus indicate that the abnormalities resulting from sustained Egr-2 expression are not due to increased apoptosis. Instead the impaired development of DP thymocytes and pro-B cells appears to be due to the blockade of the differentiation of early lineage-restricted cells into fully committed pre-B cells or DP thymocytes. One intriguing possibility is that the expression of Egr molecules may be inherited from hematopoietic stem cells and that Egr molecules may serve to support the early stages of lymphocyte development and are then downregulated before final commitment to a specific lineage.

It has been reported that Egr-1 induces expression of ID3 [20]. ID3 is one of the ID proteins that modulate function of E2A proteins [23]. The E2A transcription factors are essential for the commitment and differentiation of B- and T-lymphocytes [23]. Deficiency in the E2A pathway results in a defective transition of DN to DP stage as seen in Egr-2 cTg mice [20]. Although E-box – ID pathways may be regulated by Egr-1, the expression of ID3 is unchanged in Egr-2 expressing DN and ISP cells (data not shown) suggesting that Egr-2 does not directly induce ID3 expression.

The involvement of Egr-2 in early development of T and B cells is supported by the impaired expression of lineage specific transcription factors Notch1 in thymocytes and Pax5 in pro-B cells in Egr-2 cTg mice. Activation of the Pax5 and Notch1 pathways is essential for the lineage commitment and maturation of B and T lymphocytes, respectively [1], [2]. Pax5 is exclusively expressed in the B lymphoid lineage from the committed pro-B cells to the mature B cell stage and controls the commitment of lymphoid progenitors to the B cell pathway [2]. Notch signaling is important during the early development of thymocytes but the regulation of Notch expression is not clear [1]. Notch1 is highly expressed at early stages of DN cells and the expression is reduced after β-selection and downregulated in DP cells [23], [24]. Although both Notch1 and Egr molecules are expressed in DN cells, only Notch1 is detected in ISP cells. Interestingly, sustained expression of Egr-2 does not affect Notch1 expression in DN cells, but reduced it in ISP cells, suggesting that regulation of Notch1 by Egr-2 is conditional upon other factors and may depend upon the stage of thymocyte development. Egr-2 has been found to play different roles in macrophage differentiation based on its association with PU.1 [25]. PU.1 is expressed in the early stages of T and B cell development [1], [2], and whether PU.1 regulates the expression and function of Egr-2 in suppression of the Notch and Pax5 signaling pathways at the early stages of DN or pro-B cell development, respectively, remains to be investigated. The enhanced maturation of T, NKT and B cells in Egr-2 cTg mice could not be explained by the reduced expression of Notch1 in ISP and Pax5 in pro-B cells. The completely opposite functions of Egr-2 induced at the early and late stages of lymphocyte development suggest that the function of Egr-2 may depend on the function of stage specific transcription factors. The establishment of Egr-1, -2, -3 null and inducible Egr transgenic mice will be essential to define the redundant and specific roles of these three Egr molecules at the early and late stages of lymphocyte development and to characterize stage specific function.

## Supporting Information

Figure S1 **Expression of Egr-2 in subsets of thymocytes (A and B) and B cells (C).** Subsets of thymocytes and B cells were sorted according to surface markers as described in material and method. Total RNA was used for RT-PCR analysis. The expression was normalized against actin.
(TIF)

Figure S2 **TCR signalling is normal in thymocytes from wild type and Egr-2 cTg mice.** A). Thymocytes were isolated and stimulated with or without pre-coated anti-CD3 (5 ug/ml) for 60 minutes. Total cell lysates were immunoblotted with antibodies against pERK or ERK. B). Isolated thymocytes were plated at 5×10^5^/well in a 96-well plate pre-coated anti-CD3 and cultured for three days, then pulsed for 8 hours with ^3^H-thymidine. The incorporated of ^3^H was measured. The experiments were from thymocytes pooled from five mice.
(TIF)
