# Supplementary figures and images for: Early Growth Response Gene-2 (Egr-2) Regulates the Development of B and T Cells
Source: PLoS One. 2011 Apr 14;6(4):e18498. doi: 10.1371/journal.pone.0018498 (PMC3077377; doi:10.1371/journal.pone.0018498)

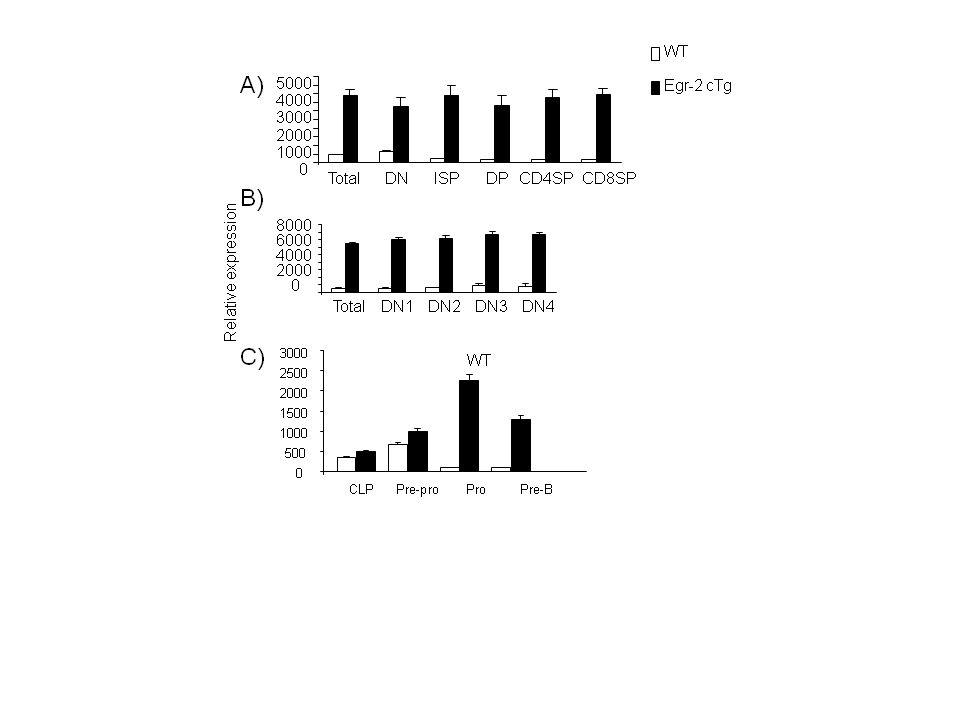

Supplement: Figure S1 — Expression of Egr-2 in subsets of thymocytes (A and B) and B cells (C). Subsets of thymocytes and B cells were sorted according to surface markers as described in material and method. Total RNA was used for RT-PCR analysis. The expression was normalized against actin. (TIF) [file pone.0018498.s001.tif]

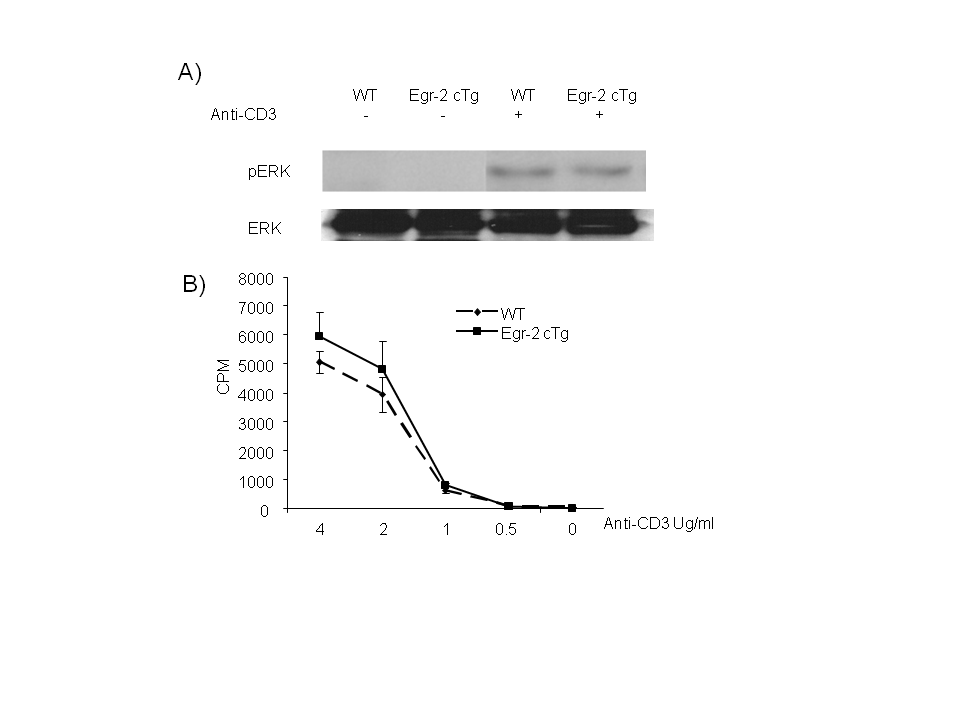

Supplement: Figure S2 — TCR signalling is normal in thymocytes from wild type and Egr-2 cTg mice. A). Thymocytes were isolated and stimulated with or without pre-coated anti-CD3 (5 ug/ml) for 60 minutes. Total cell lysates were immunoblotted with antibodies against pERK or ERK. B). Isolated thymocytes were plated at 5×10^5^/well in a 96-well plate pre-coated anti-CD3 and cultured for three days, then pulsed for 8 hours with 3H-thymidine. The incorporated of 3H was measured. The experiments were from thymocytes pooled from five mice. (TIF) [file pone.0018498.s002.tif]
